# Ostreid herpesvirus type 1 replication and host response in adult Pacific oysters, *Crassostrea gigas*

**DOI:** 10.1186/s13567-014-0103-x

**Published:** 2014-10-08

**Authors:** Amélie Segarra, Laury Baillon, Delphine Tourbiez, Abdellah Benabdelmouna, Nicole Faury, Nathalie Bourgougnon, Tristan Renault

**Affiliations:** Ifremer (Institut Français de Recherche pour l’Exploitation de la Mer), Unité Santé Génétique et Microbiologie des Mollusques (SG2M), Laboratoire de Génétique et Pathologie des Mollusques Marins (LGPMM), Avenue de Mus de Loup, 17390 La Tremblade, France; Université de Bretagne Sud (UBS), Centre d’Enseignement et de Recherche Yves Coppens, Laboratoire de Biotechnologie et Chimie Marines EA3884 (LBCM), Université Européenne de Bretagne (UEB), Campus de Tohannic, BP573, 56017 Vannes Cedex, France

## Abstract

Since 2008, massive mortality outbreaks associated with OsHV-1 detection have been reported in *Crassostrea gigas* spat and juveniles in several countries. Nevertheless, adult oysters do not demonstrate mortality in the field related to OsHV-1 detection and were thus assumed to be more resistant to viral infection. Determining how virus and adult oyster interact is a major goal in understanding why mortality events are not reported among adult Pacific oysters. Dual transcriptomics of virus-host interactions were explored by real-time PCR in adult oysters after a virus injection. Thirty-nine viral genes and five host genes including MyD88, IFI44, IkB2, IAP and Gly were measured at 0.5, 10, 26, 72 and 144 hours post infection (hpi). No viral RNA among the 39 genes was detected at 144 hpi suggesting the adult oysters are able to inhibit viral replication. Moreover, the IAP gene (oyster gene) shows significant up-regulation in infected adults compared to control adults. This result suggests that over-expression of IAP could be a reaction to OsHV-1 infection, which may induce the apoptotic process. Apoptosis could be a main mechanism involved in disease resistance in adults. Antiviral activity of haemolymph against herpes simplex virus (HSV-1) was not significantly different between infected adults versus control.

## Introduction

In 1972, virus particles morphologically similar to herpesviruses were first reported in an invertebrate, the Eastern oyster, *Crassostrea virginica*, from the USA [1]. Since then, the new family *Malacoherpesviridae* in the order *Herpesvirales* has been created in order to include a virus named ostreid herpesvirus type 1 (OsHV-1) infecting different bivalve species [2-4]. OsHV-1 is reported in mass mortality outbreaks observed among Pacific oysters less than one year old [5-8]. Considering the economic importance of shellfish culture, tools have been previously developed for rapid virus detection [5,9-12] and the OsHV-1 genome has been fully sequenced from purified virus particles [3].

Since 2008, mass mortality outbreaks (with rates of up to 80%) were reported in *C. gigas* spat and juveniles in Europe, New Zealand and Australia [13-15] and were associated with virus variants [16-18]. The role of OsHV-1 in these mortalities has been underlined by experimental infection of *C. gigas* spat with viral suspension [19,20]. These abnormal mortalities have not occurred among *C. gigas* adults in France. Nevertheless, virus DNA and proteins can be detected in adult oysters in the absence of mortality [21,22]. Similar observations were reported in vertebrate herpesviruses such as channel catfish virus (CCV) in channel catfish adults [23], or *Oncorhynchus masou* virus in salmonid adults [24]. In vertebrates, the strategies that herpesviruses use to infect cells during lytic or latent infection have been studied intensively [25]. However, Information on these aspects of infection by OsHV-1 is rudimentary.

Oysters have an efficient defense mechanism based on innate immunity. Studies have demonstrated that the immune system of *C. gigas* is capable of recognizing virus infection with resulting variations in expression of genes involved in virus recognition, signaling and immunity, that is Myeloid Differentiation factor 88, IkB2, Interferon-induced protein 44, Glypican and apoptosis inhibitor [26-29]. Due to the lack of molluscan cell lines for culturing OsHV-1, antiviral activities of mollusks are often tested against another virus, using a plaque assay [28,30-32]. *Crassostrea gigas* heamolymph contains a compound(s) that inhibits herpes simplex virus type 1 (HSV-1) replication in Vero cells [28,30,31]. To date, investigations into in vitro antiviral responses of *C. gigas* after an OsHV-1 infection have not been undertaken.

Although experimental protocols have been developed recently to reproduce the viral infection in laboratory conditions [19,20] the dynamic processes of infection have not been studied in experimental conditions at the adult stage.

In this context, adult oysters appear as animals of interest to study host and OsHV-1 interactions. Based on previous observations adult oysters are assumed to be more resistant to viral infection.

In this study, we explored what happens in adult oysters after a virus injection through monitoring mortality, antiviral activities and viral and oyster gene expression.

## Material and methods

### Animals and experimental infection design

Two families of Pacific oysters, *Crassostrea gigas,* with different genetic backgrounds were used in this study. Two families, A1 and A2, were produced using different genitors (one male and one female for each family) in the Ifremer facilities located in La Tremblade (Charente Maritime, France) in 2010. They were characterized by high survival rates during their first year of life when spat were reared in the field [33]. Experimental infections were carried out using oysters from both families (two years old, 6 cm in length for A1 and 19 months old, 4 cm in length for A2). For each family, 240 oysters were “anaesthetized” during 4 h in a solution containing magnesium chloride (MgCl_2_, 50 g/L) in seawater (1 v)/ distilled water (4 v) [34]. One hundred μL of an OsHV-1 (μVar [16]) suspension at 1.10^6^ copies of viral DNA/μL were injected into the adductor muscle of 120 oysters per family [19,20]. Similarly, 120 adults from each family were injected with 100 μL of sterile artificial seawater as controls. Pacific oysters were then placed in four tanks (30 oysters per tank/condition) containing 5 L of filtered seawater (1 μm) at 22 °C. One tank per condition was dedicated to survival record and the three other were dedicated to sample collection. The absence of bacterial contamination of the viral suspension was tested before each challenge. The viral suspension was spread onto a Petri dish which contained marine agar and no bacteria were observed.

Twenty oyster spat (7 months old, 3 cm in length) were simultaneously injected in order to confirm that the virus suspension was infectious and mortality was monitored without sample collection. Spat were produced at the Ifremer facilities (La Tremblade, Charente Maritime, France) from wild oysters which were collected from the Marennes-Oléron Bay in January 2011.

Mortality was monitored during a period of 144 h and cumulative mortality was defined daily for each condition (oysters infected with OsHV-1 or injected with sterile artificial seawater).

### Plasma and tissue sample collection

For both families (A1 and A2), haemolymph was collected from six live oysters with a sterile syringe (1 ml, Terumo) from the pericardial cavity and pooled for each tank, condition and family. These samples were collected at 0.5, 10, 26, 72 and 144 hours post-infection (hpi). Haemolymph pools were centrifuged at 300 rpm during 10 min at 4 °C then filtered at 0.22 μm in order to study antiviral activity. All plasma samples (without haemocytes) were stored at −80 °C.

Two pieces of mantle were sampled from two live individuals per tank (6 individuals per time/condition) from both families. A piece of mantle (50 to 100 mg) was disposed in a tube containing 1 mL of TRIZOL® Reagent™ (Ambion®) and frozen at −80 °C for further RNA extraction. The second piece of mantle was directly frozen at −20 °C for DNA extraction.

### DNA and RNA extraction and cDNA synthesis

DNA extraction was performed using a QiAamp tissue mini kit® (QIAgen) according to the manufacturer’s protocol. Elution was performed in 100 μL of AE buffer provided in the kit.

Total RNA was extracted using TRIZOL® Reagent™ (Ambion®) according to the manufacturer’s recommendation. Total RNA was treated with Turbo™ DNAse (Ambion®) to remove genomic DNA. The RNA quality and quantity were determined using NanoDrop 2000 (Thermo Scientific) and Bioanalyser 2100 (Agilent). First-strand cDNA synthesis was carried out using the SuperScript ® III First-Strand Synthesis System (Invitrogen) using 8000 ng of treated RNA. Reactions lacking RT were performed after each DNAse treatment using real-time PCR in order to control the absence of oyster and/or virus genomic DNA.

### OsHV-1 DNA quantification

Viral DNA quantification was carried out by real-time PCR in duplicate using a Mx3000 Thermocycler (Agilent) according to previously described protocols [11,35]. Amplification reactions were performed in a total volume of 20 μL to study oyster gene or viral gene expression. Each well contained 5 μL of DNA, 10 μL of Brillant III Ultra-Fast SYBR® Green PCR Master Mix (Agilent), 2 μL of each primer (3 μM) and 1 μL of distilled water. Real time PCR cycling conditions were as follow: 3 min at 95 °C, followed by 40 cycles of amplification at 95 °C for 5 s, 60 °C for 20 s. Melting curves were plotted (55–95 °C) in order to ensure that a single PCR product was amplified for each set of primers. Negative controls (without DNA) were included to rule out DNA contamination.

### Gene expression analysis

Real-time RT PCR was used to follow 39 viral genes [35] and five host genes. In both cases, the protocol was the same as that previously described with 5 μL of cDNA dilution (1/30) instead of genomic DNA.

#### Viral gene expression

Viral RNA detection was performed only for family A2. Normalized relative viral gene expression levels were calculated for each individual using the formula: F = log10[(E + 1)^40-Ct^/N] adapted from de Decker et al. [36], where E is efficiency of each primer couple [35], Ct (threshold cycle) corresponds to the PCR cycle number, N is the maximal number of viral DNA copies/μL of total DNA detected minus the number of viral DNA copies/μL of total DNA determined by absolute real time PCR for each individual and Ct = 40 is arbitrarily considered to correspond to “no Ct” obtained by real-time PCR.

#### Host gene expression

Expression of five oyster immune related genes was monitored only from family A2 by real-time PCR: myeloid differentiation factor 88 (MyD88), NF kappa-B inhibitor cactus (IKB2), interferon-induced protein 44 (IFI44), glypican (Gly) and Baculoviral apoptosis inhibitor repeat-containing protein 2 (IAP) genes [29]. The relative quantification value (ratio R) was calculated as described in Segarra et al. [29] using the method described by Pfaffl [37].

Host gene expression was normalized to that of elongation factor I (EF), as no significant differences of Ct values were observed for this housekeeping gene between several conditions during the course time (CV = 2.2%, Kruskal-Wallis test Z = 0.10, *p* > 0.9). The calibrator used for the experiment was individual sampling at time 0.5 hpi for family A2.

### Cytotoxicity and antiviral activity of haemolymph against HSV-1

Cytotoxicity and antiviral activity of oyster haemolymph were monitored in vitro on Vero cells as previously described [30,38]. HSV-1 titration was performed by the limiting dilution method [39]. The HSV-1 stock had a titer of 2.10^7,4^ ID_50_/mL and MOI was 0.001 ID_50_/cell. One hundred μL of African green monkey kidney cell suspension (3.5 × 10^5^ Vero ATCC CCL-81 cells/mL) diluted with MEM 8% FCS were distributed into the wells of a 96 well plate. Cell suspensions were incubated with 50 μL of oyster haemolymph and/or 50 μL of HSV-1 cell suspension (48, 72, and 90 h, 37 °C, 5% CO2) in Eagle’s MEM containing 8% FCS. Triplicates of haemolymph suspension were tested. This model is currently used for the screening of antiviral molecules from marine organisms [40]. After 48, 72 and 96 h of incubation, antiviral activity was evaluated by the neutral red dye method [41]. After shaking, optical density (OD) was measured at 540 nm using a spectrophotometer (SpectraCountTM; Packard). Cell and HSV-1 controls were run simultaneously. The antiherpetic compound acyclovir [9-(2-hydroxyethoxymethyl) guanine] (Merck), 25 mg/mL was used as a reference for HSV-1 inhibition.

The percentage of cytotoxicity was calculated as [(OD)C − (OD)Oyster/(OD)C] × 100 where (OD)C and (OD)Oyster were the OD values of the control cells (without haemolymph or HSV-1) and treated cells with haemolymph, respectively [42]. The antiviral activity was calculated by: [((OD)OysterHSV-1 − (OD)HSV-1)/((OD)Oyster − (OD)HSV-1)] × 100 where, (OD)OysterHSV-1 was the OD of the test haemolymph with HSV-1, (OD)HSV-1 was OD of HSV-1 infected control cells, (OD)Oyster was the OD of the test haemolymph.

### Data analysis

Kaplan-Meier survival curves and the Wilcoxon test were used to characterize and compare survival between adult oyster families A1 and A2, and spat.

CT were calculated with the Stratagene Mxpro software 4.0. A heatmap was generated in order to represent the viral gene expression using the “heatmap” package in R. Clustering of viral genes was performed using the euclidean method. Results for relative expression of oyster genes were expressed as mean ± standard error. A Mann–Whitney test was used to analyze oyster gene expression.

Correlations between viral DNA amounts and antiviral activity of haemolymph were tested with Spearman’s nonparametric rho using the XLSTAT software (version 2013), and the results were declared statistically significant at the two-sided 5% (i.e., *P* < 0.05).

## Results

### Mortality and virus DNA detection for A1 and A2 families

**Figure 1:**
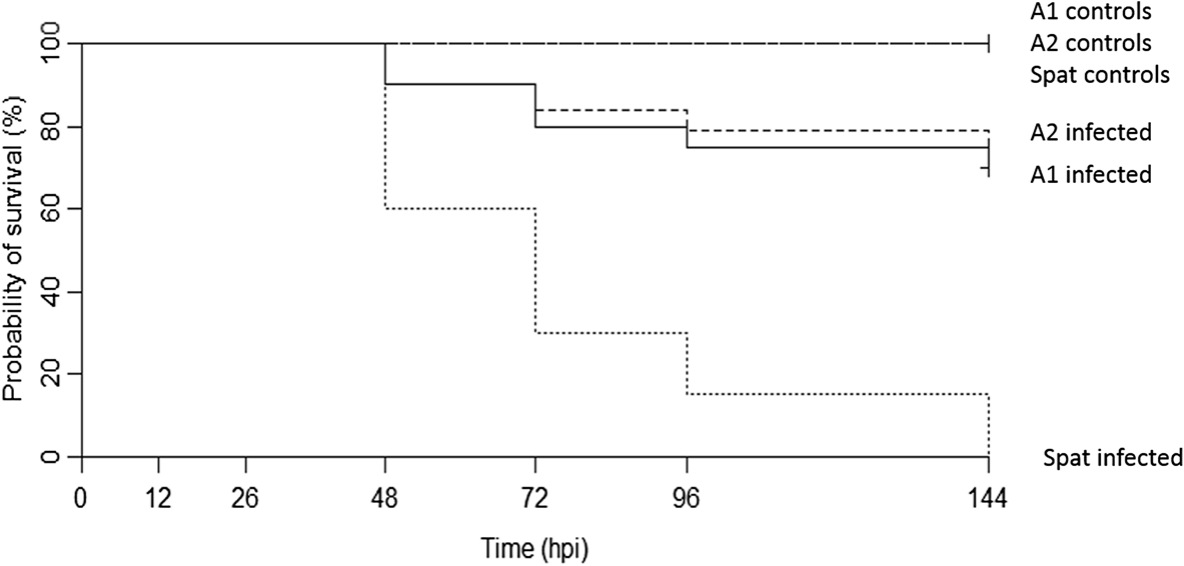
**Survival of** ***Crassostrea gigas*** **adult oysters, A1 and A2 families, versus spat oysters during an experimental infection with OsHV-1.** *n* = 30 oysters/condition. Controls were injected with artificial seawater.

The respective mean survival rates of families A1 and A2 were 70% and 75% at 144 hpi (Kaplan-Meier, Figure 1). The mean survival rates for oyster spat injected with the same viral suspension was 0% at 144 hpi (Figure 1). Mortality rates of infected adults (A1 and A2) were significantly different from controls and infected spat (Wilcoxon test, *p* < 0.05).

**Figure 2:**
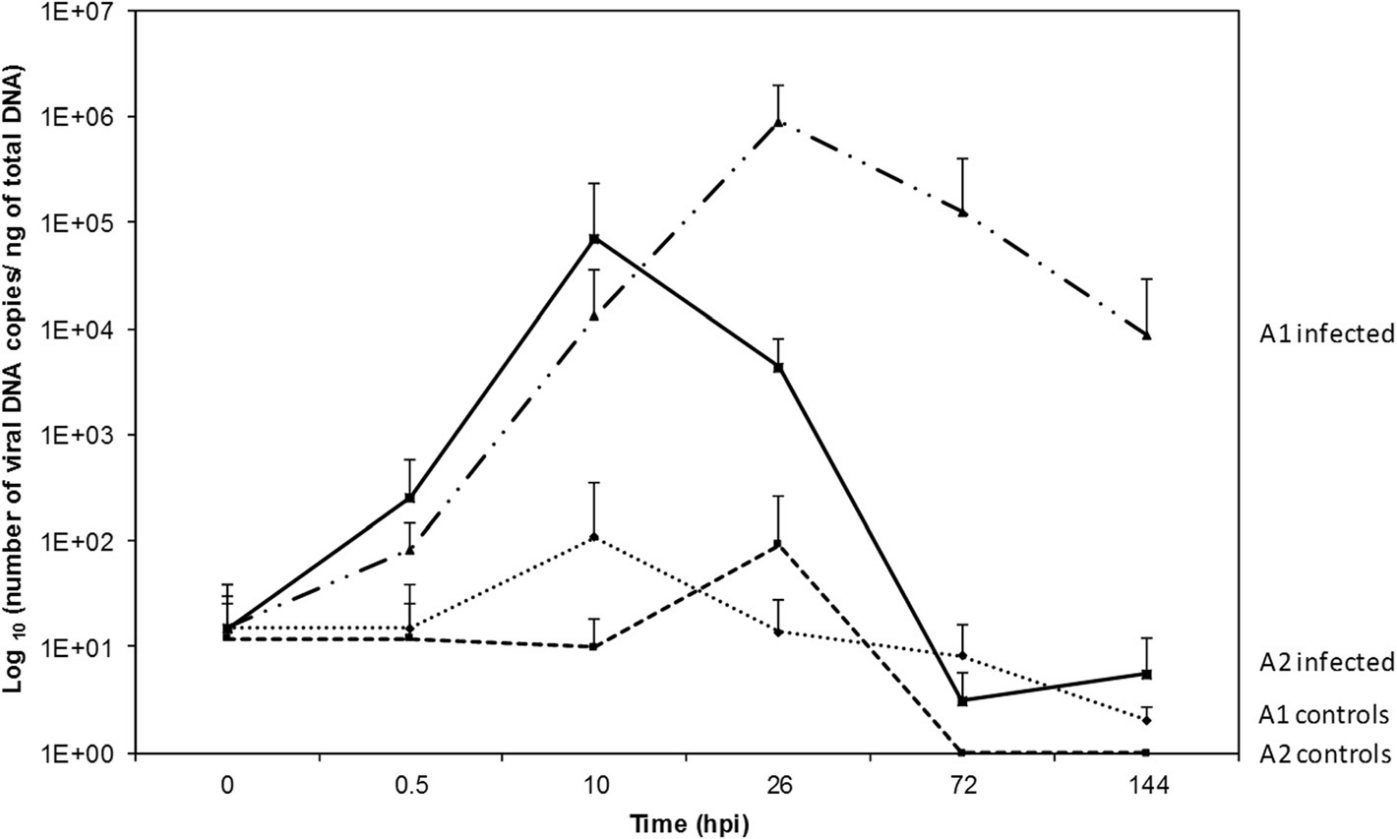
**Virus DNA detection curves by real time quantitative PCR in adult oyster families A1 and A2.** Average *n* = 6. Error bars represent ± standard deviation.

Although no mortality was observed in control adult oysters (oysters injected with sterile seawater), viral DNA was detected by real-time PCR in animals from both families (A1 and A2) (Figure 2). For control adults, the mean OsHV-1 DNA amounts were 1.5 × 10^1^ viral DNA copies/ng of total DNA at 0.5 hpi from both families. Viral amounts increased and peaked at 10 hpi (1.0 × 10^2^ viral DNA copies/ng of total DNA) and 26 hpi (9.1 × 10^2^ viral DNA copies/ng of total DNA) in control adults from families A1 and A2, respectively (Figure 2) and decreased after these times. At later time points, the mean viral DNA amount observed was estimated at less than 10 viral DNA copies/ng of total DNA in A1 and A2 controls (Figure 2).

Concerning adults injected with the OsHV-1 suspension, viral DNA amounts increased to reach 7.1 × 10^4^ viral DNA copies/ng of total DNA at 10 hpi and 8.9 × 10^5^ viral DNA copies/ng of total DNA at 26 hpi in A2 and A1, respectively (Figure 2). At the end of the challenge, the viral DNA amounts were significantly different (*p* < 0.005) between both families. The viral amounts decreased during the challenge to reach < 10 and 8.8 × 10^3^ viral DNA copies/ng of total DNA at 144 hpi in A2 and A1, respectively (Figure 2).

### Dual transcriptomics of OsHV-1 and oyster genes in the family A2

**Figure 3:**
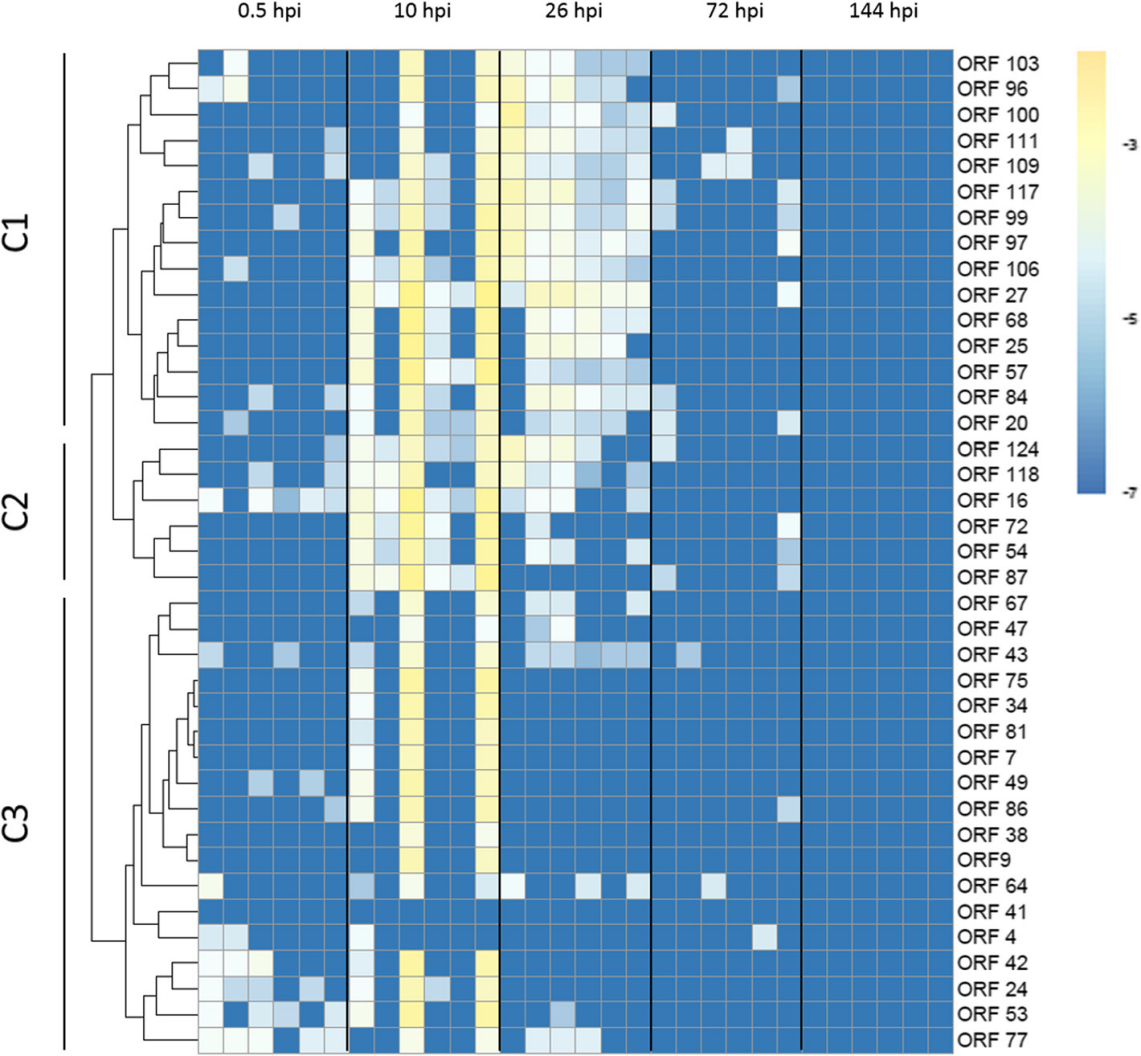
**Heatmap illustrating the viral expression levels of 39 genes of OsHV-1 at different post infection time points in family A2.** C1, C2 and C3 correspond to clusters of viral genes. Colors represent the fold change, blue and yellow denote low and high expression respectively. Rows are 39 viral genes and each column represents an individual (*n* = 6 per time). The full list of genes is available in Segarra et al. [29].

Family A2 was selected in view of few viral DNA amounts detected at the end of the challenge. The expression levels of 39 OsHV-1 genes were studied by real-time PCR in the mantle. The 39 selected genes were selected in previous studies based on the literature and/or their predicted functions [3,35]. The fold change of −7 (blue) consisted of no viral transcript detection, while −3 (yellow) consisted of highly expressed genes. No viral RNA was detected before the experimental infection (data not shown) and the first viral RNA was observed at 0.5, 10, 26 and 72 hpi (Figure 3).

**Figure 4:**
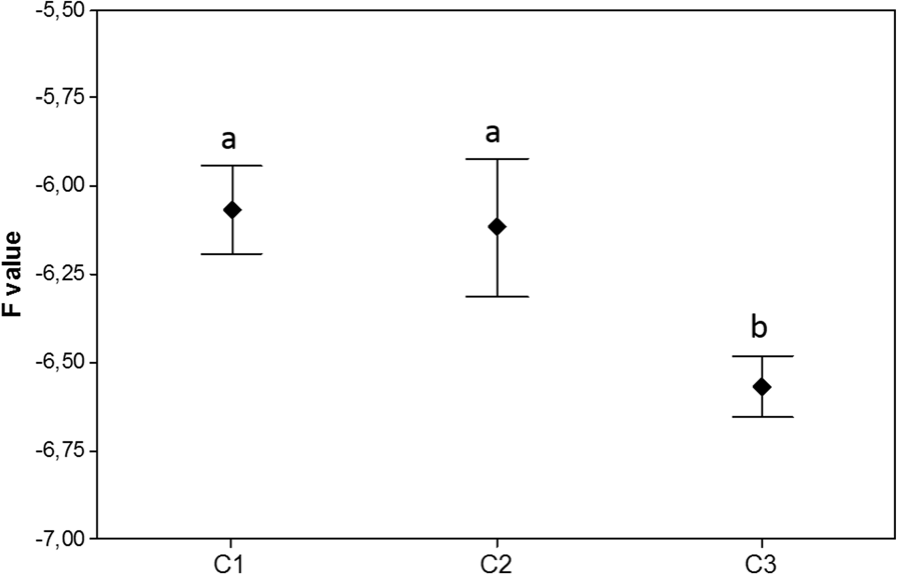
**Means of viral gene expression for each cluster.** Different lowercase letters indicate significant differences (Mann–Whitney test, *p* < 0.05) between clusters. Error bars represent ± standard deviation.

Differences in terms of gene expression levels were observed between individuals collected at the same time. Although few transcripts were detected at 0.5 and 72 hpi, we observed clearly an up regulation of almost all virus genes in three individuals at 10 hpi (Fold change = −3) (Figure 3). About half of the viral genes were expressed at 26 hpi in all individuals. Finally, no transcripts were detected in all individuals at 144 hpi (Figure 3). Virus transcripts were grouped by the Euclidean method to produce a dendrogram comparing the expression levels of the 39 ORF. Three clusters were observed: one group of highly expressed genes (C1), one group of moderately expressed genes (C2) and one group of low expressed genes (C3) (Figure 4). No significant difference was observed between C1 and C2 clusters. Nevertheless, genes included in the C3 cluster were significant up-regulated compared to other genes (Mann–Whitney, *p* < 0.05) (Figure 4).

**Figure 5:**
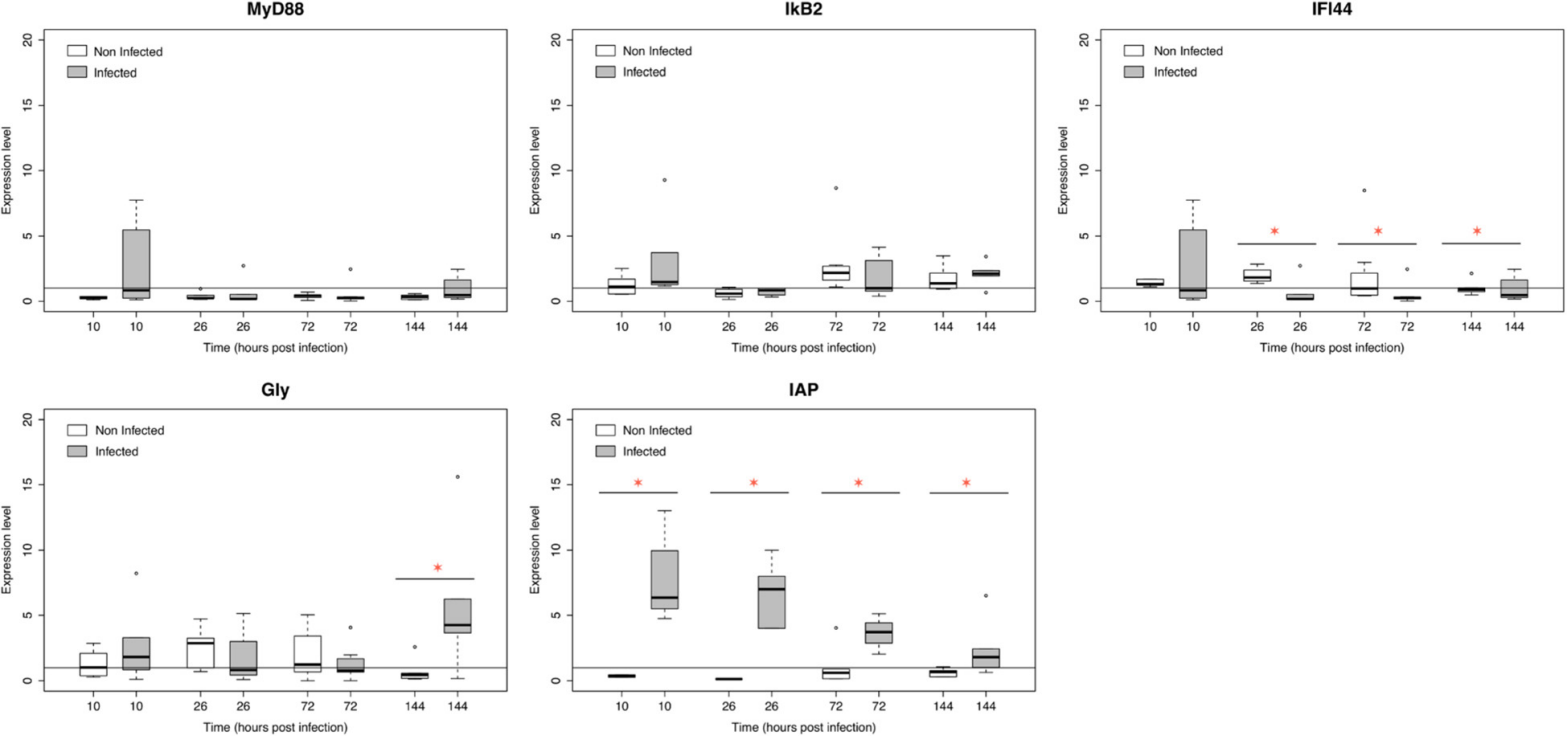
**Relative expression for family A2 by real time PCR of five selected oyster genes.** (MyD88: myeloid differentiation factor 88, IkB2: Inhibitor of nuclear factor kappaB kinase beta, IFI44: Interferon induced protein 44, Gly: Glypican and IAP: Inhibitor of apoptosis) at 10, 26, 72 and 144 hpi. White boxplots: non-infected oysters; grey boxplots: infected oysters. Expression levels were normalized to EF (mean ± SD, *n* = 6). Controls are arbitrarily assigned to a value of 1. *Significant difference of gene expression compared to controls by Mann–Whitney test.

Concerning the five selected oyster genes, only three showed significantly different expressions between control adults and oysters injected with OsHV-1: IFI44 at 26, 72 and 144 hpi, Glypican at 144 hpi, and IAP at 10, 26, 72 and 144 hpi (Figure 5). Among these three genes, only IFI44 gene expression was down regulated at 26, 72 and 144 hpi in infected oysters compared to controls (*p* < 0.05). The two other genes were over-expressed. The IAP expression level decreased during the experimental infection but remained up regulated in infected oysters compared to controls (Figure 5).

### Antiviral activity of haemolymph against HSV-1

**Figure 6:**
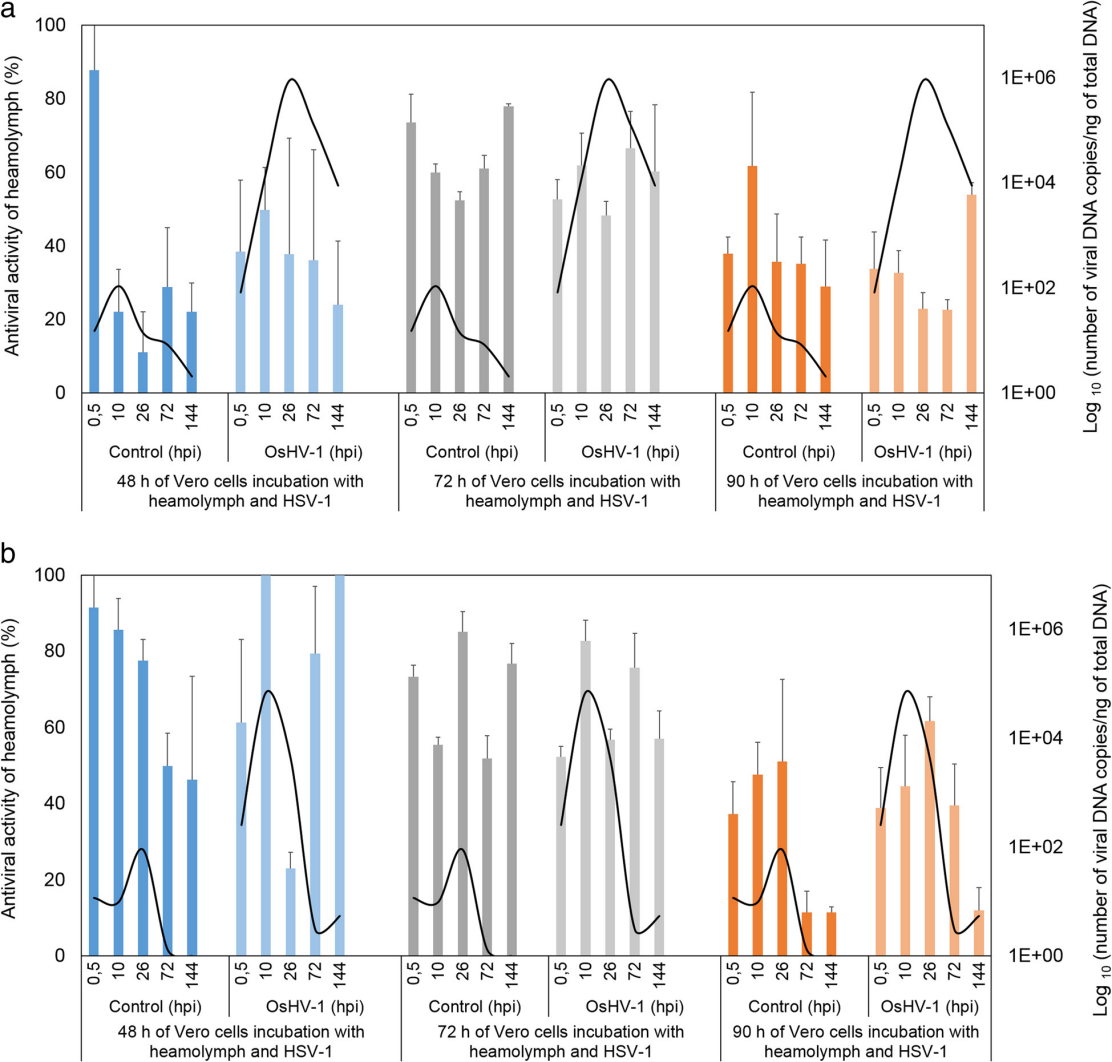
**Antiviral activity kinetic of adult oyster haemolymph stimulated or not stimulated with OsHV-1 for family A1 (a) and family A2 (b) at different times in relation to HSV-1 infection of Vero cells.** Blue bars, 48 h of Vero cell incubation with heamolymph and HSV-1 virus; Grey bars, 72 h of Vero cell incubation with heamolymph and HSV-1 virus and orange bars, 90 h of Vero cell incubation with heamolymph and HSV-1 virus. The viral DNA quantification is presented by black curves.

Acyclovir (5 and 1 μg/mL) conferred high protection (95%) against HSV-1 with a low cell destruction (10%) (data not shown). For both families, antiviral activity of haemolymph with or without OsHV-1 stimuli was not significantly different at 48, 72 or 90 h incubation of Vero cells (Figures 6A and B). Moreover, the average cytotoxicity activity ranged from 20 to 35% for both families (data not shown). The lowest cytotoxicity rate was observed for a cell incubation period of 48 h in family A2. Positive correlation between viral DNA amounts and antiviral activity was observed only for control and infected oysters with OsHV-1 in family A2 after 90 h of Vero cell incubation (rho = 0.7, *p* < 0.05) (Figure 6B).

## Discussion

Adult oysters do not demonstrate mortality in the field related to OsHV-1 detection. They were thus assumed to be more resistant than juveniles to viral infection [21,43]. However as Pacific oysters do not show macroscopic symptoms before dying during the course of OsHV-1 infection, it remains difficult in the absence of mortality reports to know whether adults are susceptible to infection. Searching for viral DNA, RNA and proteins is one way to explore this question. Although Arzul et al. [21] demonstrated that viral DNA and proteins could be detected in apparently healthy adult oysters collected in the field, the development of the viral infection has never been studied in experimental conditions in the adult stage. An experimental protocol has been recently developed to reproduce the viral infection in oyster spat [19]. This protocol was used in the present study to explore what happened in adult oysters. Adults appear as a development stage of interest to inform (i) if the host is less susceptible at the adult stage than at younger stages, (ii) how adult oysters are able to manage the viral infection or (iii) what type of infection occurs in adults (i.e. acute versus persistent infection).

Recently, a study was performed to follow OsHV-1 gene expression in oyster spat [35]. Authors showed that viral transcripts were less expressed after 72 hpi in less susceptible oysters compared to highly susceptible ones [29]. Here, similar results were observed in adults after injection of a virus suspension with a prior increase of OsHV-1 DNA amounts and viral RNA expression followed by decreases at 72 hpi and 144 hpi. These results suggested that the virus was able to replicate in adult oysters. However, adult oysters also demonstrated a strong ability to control the viral infection in the tested conditions. In the present study, we report a potentially reproducible experimental model of OsHV-1 infection in adult Pacific oysters. This experimental model described shows that, during the acute phase of OsHV-1 infection, multiple viral genes were abundantly expressed resulting in large amounts of viral DNA and associated mortality in infected oysters. However after the acute phase, viral DNA was present but with undetectable viral gene expression. Moreover, the amounts of viral DNA detected were much lower than those detected in the acute phase and no mortality was recorded. These results suggest that OsHV-1 DNA could be maintained at a low copy number during the phase following the acute phase of infection. This has previously been reported for persistent (and latent) infections in other herpesviruses [44].

Moreover, adult oysters used in the present study were positive for OsHV-1 DNA detection before the virus challenge. It consolidates the idea that adults may play the role of reservoirs for OsHV-1 as previously hypothesized [21,45].

Questions were thus opened about the status of the virus in adult oysters: (i) low level of virus replication related to an effective immunity or (ii) true latency involving the expression of a small number of viral genes.

Three hypotheses could be proposed to explain the lack of detection of viral transcripts in adults before the virus challenge and at 144 hpi. Firstly, the single tissue selected in the present study (mantle) to detect OsHV-1 RNA may not be the best one to search for viral RNA in cases of chronic infection related to low virus replication or true latency. Although the mantle has been previously demonstrated as a site of high replication in naturally and experimentally infected spat [10,19,20], few data are available for adult oysters. Vertebrate herpesviruses are able to maintain more than one type of persistent infection at the same time and in different cell types. For example, chronic infection by the Epstein-Barr virus (EBV) has been observed in pharyngeal epithelial cells whereas in the same individual it latently infected B lymphocytes [46,47] and herpes simplex virus (HSV) can undergo a lytic or latent infection in different cell types [48]. Therefore, in one individual, persistent infection with a single virus may involve multiple types of persistence.

Secondly, 39 viral genes were selected among the 124 from the virus genome and we could have missed those mainly involved in true latency. Finally, viral multiplication or latency could be related to levels of virus gene expression below the threshold of detection by real-time PCR. In HIV infection, antiviral treatment can reduce plasma and lymph node RNA levels below the level of detection of sensitive assays for nucleic acid [49].

Studies have demonstrated that the immune system of *C. gigas* spat is capable of recognizing virus infection with resulting variations in expression of genes involved in virus recognition, signaling and immunity such as Myeloid Differentiation factor 88, IkB2, Interferon-induced protein 44, Glypican and apoptosis inhibitor [26-29]. In the current study, only three of the five selected oyster genes showed significant differences between experimentally infected oysters and control ones: IFI44, Glypican and IAP. Previous research has demonstrated that IFI44, a member of the IFN inducible gene family, was strongly up-regulated in heamolymph of infected juvenile oysters at 24 and 48 hpi [26], and in the mantle of infected spat oysters at 4, 12, 26, 72 and 144 hpi [31]. Here, IFI44 was significantly down-regulated in adults after virus injection at 26, 72 and 144 hpi compared to control adults. Differences may be related to the operation of different defense mechanisms depending on the development stage, spat versus adults. Moreover, Pacific oysters studied in the present study were identified as infected (viral DNA detection) before experimental infection and it is possible that the immune responses to OsHV-1 infection differ in primary infection and reinfection. Results reported concerning MyD88 expression were in accordance with results previously reported in experimentally infected spat [29]. In vertebrates, this gene was described as an essential adaptor protein in the Toll/IL-1 receptor family signaling pathways [50]. Although MyD88 gene was demonstrated to be up-regulated during OsHV-1 infection before mortality occurred [26,51], Segarra et al. [29] showed no significant difference concerning MyD88 expression level between surviving spat after virus injection and control oysters at 72 and 144 hpi. These results suggested that up-regulation of MyD88 gene is more a marker of infection than a marker of effective immunity.

Our data also show that Glypican was up-regulated in infected oysters versus controls only at 144 hpi. Segarra et al. [29] previously reported that Glypican expression was also up-regulated in surviving oyster spat at 144 hpi compared to controls. Glypicans belong to a family of heparan sulfate proteoglycans that are linked to the cell surface by a glycosylphosphatidylinositol (GPI) anchor. Glypicans play important roles in cellular growth, development [52], proteolysis and apoptosis [53,54].

In agreement with previous studies [29], our results also show that the IAP gene was up-regulated during viral infection compared to controls. Over-expression of IAP could be a reaction to the apoptotic process induced by OsHV-1 infection. The capacity of adult oysters to inhibit and control the apoptotic process could be essential for survival. Indeed, it has been proposed that high mortality rates affecting shrimp during viral infections could be related to a non-regulated apoptosis process [55].

Future research based on protein expression is now needed to confirm these results. Another experiment is currently in progress in the laboratory to analyze expression and tissue distribution of viral and host proteins using specific antibodies. In the current study, we measured antiviral activity of the haemolymph in adults after OsHV-1 injection in order to stimulate the antiviral response and the production of antiviral molecules. We can presume that adult oysters are able to maintain an effective immunity through antiviral activities. Since no marine bivalve cell lines are available, studies have evaluated antiviral activity in aquatic invertebrates using heterologous cell lines including Vero cells [30,32,56-58]. Other studies [28,30,31,59,60] reported the presence of antiviral activities against different viruses in haemolymph of marine molluscs including the Pacific oyster, *C. gigas*, and showed higher anti-HSV-1 activity during the summer [30,61]. Here, we observed a positive correlation between viral DNA amounts and antiviral activity levels at 90 h of incubation from both conditions for family A2. Viral activities observed in control animals could be explained by OsHV-1 infection before challenge.

We can assume that the heterologous model needs to be improved in order to obtain a suitable model for screening oysters for potential antiviral activities against OsHV-1. For the first time, mortality was reported in adults in experimental conditions. We show that OsHV-1 was able to replicate at this stage of development. The decrease of viral transcript levels during the course of the assay suggests that adult oysters were able to manage the viral infection and control its replication.

## References

[1] Farley CA, Banfield WG, Kasnic G, Foster WS (1972). Oyster herpes-type virus. Science.

[2] Le Deuff RM, Renault T (1999). Purification and partial genome characterization of a herpes-like virus infecting the Japanese oyster, Crassostrea gigas. J Gen Virol.

[3] Davison AJ, Trus BL, Cheng N, Steven AC, Watson MS, Cunningham C, Le Deuff RM, Renault T (2005). A novel class of herpesvirus with bivalve hosts. J Gen Virol.

[4] Davison AJ, Eberle R, Ehlers B, Hayward GS, McGeoch DJ, Minson AC, Pellett PE, Roizman B, Studdert MJ, Thiry E (2009). The order Herpesvirales. Arch Virol.

[5] Renault T, Le Deuff RM, Cochennec N, Chollet B, Maffart P (1995). Herpes-like viruses associated with high mortality levels in larvae and spat of Pacific oysters, Crassostrea gigas: a comparative study, the thermal effects on virus detection in hatchery-reared larvae, reproduction of the disease in axenic larvae. Vet Res.

[6] Burge CA, Griffin FJ, Friedman CS (2006). Mortality and herpesvirus infections of the Pacific oyster Crassostrea gigas in Tomales Bay, California, USA. Dis Aquat Organ.

[7] Sauvage C, Pépin JF, Lapègue S, Boudry P, Renault T (2009). Ostreid herpes virus 1 infection in families of the Pacific oyster, Crassostrea gigas, during a summer mortality outbreak: differences in viral DNA detection and quantification using real-time PCR. Virus Res.

[8] Garcia C, Thébault A, Dégremont L, Arzul I, Miossec L, Robert M, Chollet B, François C, Joly J-P, Ferrand S, Kerdudou N, Renault T (2011). Ostreid herpesvirus 1 detection and relationship with Crassostrea gigas spat mortality in France between 1998 and 2006. Vet Res.

[9] Le Deuff RM, Renault T, Cochennec N (1995). Antibodies specific for channel catfish virus cross-react with Pacific oyster, Crassostrea gigas, herpes-like virus. Vet Res.

[10] Lipart C, Renault T (2002). Herpes-like virus detection in infected Crassostrea gigas spat using DIG-labelled probes. J Virol Methods.

[11] Pepin JF, Riou A, Renault T (2008). Rapid and sensitive detection of ostreid herpesvirus 1 in oyster samples by real-time PCR. J Virol Methods.

[12] Abend JR, Ramalingam D, Kieffer-Kwon P, Uldrick TS, Yarchoan R, Ziegelbauer JM (2012). Kaposi’s sarcoma-associated herpesvirus microRNAs target IRAK1 and MYD88, two components of the toll-like receptor/interleukin-1R signaling cascade, to reduce inflammatory-cytokine expression. J Virol.

[13] EFSA Panel on Animal Health and Welfare (AHAW) (2010). Scientific Opinion on the increased mortality events in Pacific oyster (*Crassostrea gigas*). EFSA J.

[14] Renault T, Qin JG (2012). Pacific Cupped Oyster, Crassostrea gigas, Mortality outbreaks and infectious diseases. Oysters Physiol Ecol Distrib Mortal.

[15] Jenkins C, Hick P, Gabor M, Spiers Z, Fell SA, Gu X, Read A, Go J, Dove M, O’Connor W, Kirkland PD, Frances J (2013). Identification and characterisation of an ostreid herpesvirus-1 microvariant (OsHV-1 μ-var) in Crassostrea gigas (Pacific oysters) in Australia. Dis Aquat Organ.

[16] Segarra A, Pépin JF, Arzul I, Morga B, Faury N, Renault T (2010). Detection and description of a particular Ostreid herpesvirus 1 genotype associated with massive mortality outbreaks of Pacific oysters, Crassostrea gigas, in France in 2008. Virus Res.

[17] Martenot C, Oden E, Travaillé E, Malas J-P, Houssin M (2011). Detection of different variants of Ostreid Herpesvirus 1 in the Pacific oyster, Crassostrea gigas between 2008 and 2010. Virus Res.

[18] Renault T, Moreau P, Faury N, Pepin J-F, Segarra A, Webb S (2012). Analysis of clinical ostreid herpesvirus 1 (Malacoherpesviridae) specimens by sequencing amplified fragments from three virus genome areas. J Virol.

[19] Schikorski D, Renault T, Saulnier D, Faury N, Moreau P, Pépin J-F (2011). Experimental infection of Pacific oyster Crassostrea gigas spat by ostreid herpesvirus 1: demonstration of oyster spat susceptibility. Vet Res.

[20] Schikorski D, Faury N, Pepin JF, Saulnier D, Tourbiez D, Renault T (2011). Experimental ostreid herpesvirus 1 infection of the Pacific oyster Crassostrea gigas: Kinetics of virus DNA detection by q-PCR in seawater and in oyster samples. Virus Res.

[21] Arzul I, Renault T, Thébault A, Gérard A (2002). Detection of oyster herpesvirus DNA and proteins in asymptomatic Crassostrea gigas adults. Virus Res.

[22] Barbosa-Solomieu V, Miossec L, Vázquez-Juárez R, Ascencio-Valle F, Renault T (2004). Diagnosis of Ostreid herpesvirus 1 in fixed paraffin-embedded archival samples using PCR and in situ hybridisation. J Virol Methods.

[23] Wise JA, Harrell SF, Busch RL, Boyle JA (1988). Vertical transmission of channel catfish virus. Am J Vet Res.

[24] Kimura T, Yoshimizu M, Tanaka M, Sannohe H (1981). Studies on a new virus (OMV) from Oncorhynchus masou. I. Characteristics and pathogenicity. Fish Pathol.

[25] Honess RW, Roizman B (1974). Regulation of herpesvirus macromolecular synthesis I. Cascade regulation of the synthesis of three groups of viral proteins 1. J Virol.

[26] Renault T, Faury N, Barbosa-Solomieu V, Moreau K (2011). Suppression substractive hybridisation (SSH) and real time PCR reveal differential gene expression in the Pacific cupped oyster, Crassostrea gigas, challenged with Ostreid herpesvirus 1. Dev Comp Immunol.

[27] Green TJ, Montagnani C (2013). Poly I:C induces a protective antiviral immune response in the Pacific oyster (Crassostrea gigas) against subsequent challenge with Ostreid herpesvirus (OsHV-1 μvar). Fish Shellfish Immunol.

[28] Green TJ, Montagnani C, Benkendorff K, Robinson N, Speck P (2014). Ontogeny and water temperature influences the antiviral response of the Pacific oyster, Crassostrea gigas. Fish Shellfish Immunol.

[29] Segarra A, Mauduit F, Faury N, Trancart S, Dégremont L, Tourbiez D, Haffner P, Barbosa-Solomieu V, Pépin J-F, Travers M-A, Renault T (2014). Dual transcriptomics of virus-host interactions: comparing two Pacific oyster families presenting contrasted susceptibility to ostreid herpesvirus 1. BMC Genomics.

[30] Olicard C, Didier Y, Marty C, Bourgougnon N, Renault T (2005). In vitro research of anti-HSV-1 activity in different extracts from Pacific oysters Crassostrea gigas. Dis Aquat Organ.

[31] Olicard C, Renault T, Torhy C, Benmansour A, Bourgougnon N (2005). Putative antiviral activity in hemolymph from adult Pacific oysters, *Crassostrea gigas*. Antiviral Res.

[32] Dang VT, Benkendorff K, Speck P (2011). In vitro antiviral activity against herpes simplex virus in the abalone Haliotis laevigata. J Gen Virol.

[33] Degremont L, Benabdelmouna A: **Mortality associated with OsHV-1 in spat Crassostrea gigas: role of wild-caught spat in the horizontal transmission of the disease.** *Aquac Int* 2014, in press.

[34] Namba K, Kobayashi M, Aida S, Uematsu K, Yoshida M, Kondo Y, Miyata Y (1995). Persistent relaxation of the adductor muscle of oyster Crassostrea gigas induced by magnesium ion. Fish Sci.

[35] Segarra A, Faury N, Pépin J-F, Renault T (2014). Transcriptomic study of 39 ostreid herpesvirus 1 genes during an experimental infection. J Invertebr Pathol.

[36] De Decker S, Reynaud Y, Saulnier D (2013). First molecular evidence of cross-species induction of metalloprotease gene expression in Vibrio strains pathogenic for Pacific oyster Crassostrea gigas involving a quorum sensing system. Aquaculture.

[37] Pfaffl MW (2001). A new mathematical model for relative quantification in real-time RT–PCR. Nucleic Acids Res.

[38] Defer D, Bourgougnon N, Fleury Y (2009). Screening for antibacterial and antiviral activities in three bivalve and two gastropod marine molluscs. Aquaculture.

[39] Reed LJ, Muench H (1938). A simple method of estimating fifty per cent endpoints. Am J Epidemiol.

[40] Yasin B, Pang M, Turner JS, Cho Y, Dinh NN, Waring AJ, Lehrer RI, Wagar EA (2000). Evaluation of the inactivation of infectious Herpes simplex virus by host-defense peptides. Eur J Clin Microbiol Infect Dis.

[41] McLaren C, Ellis MN, Hunter GA (1983). A colorimetric assay for the measurement of the sensitivity of herpes simplex viruses to antiviral agents. Antiviral Res.

[42] Takeuchi H, Baba M, Shigeta S (1991). An application of tetrazolium (MTT) colorimetric assay for the screening of anti-herpes simplex virus compounds. J Virol Methods.

[43] Renault T (2010). Maîtriser les maladies infectieuses pour une aquaculture durable: les maladies infectieuses chez les mollusques, un risque à maîtriser pour une aquaculture durable.

[44] Sawtell NM (1997). Comprehensive quantification of herpes simplex virus latency at the single-cell level. J Virol.

[45] Le Deuff RM, Renault T, Gerard A (1996). Effects of temperature on herpes-like virus detection among hatchery-reared larval Pacific oyster Crassostrea gigas. Dis Aquat Organ.

[46] Thompson MP, Kurzrock R (2004). Epstein-Barr virus and cancer. Clin Cancer Res.

[47] Middeldorp JM, Brink AATP, Brule VD JCA, Meijer CJLM (2003). Pathogenic roles for Epstein–Barr virus (EBV) gene products in EBV-associated proliferative disorders. Crit Rev Oncol Hematol.

[48] Knipe DM, Cliffe A (2008). Chromatin control of herpes simplex virus lytic and latent infection. Nat Rev Microbiol.

[49] Cavert W, Notermans DW, Staskus K, Wietgrefe SW, Zupancic M, Gebhard K, Henry K, Zhang Z-Q, Mills R, McDade H, Goudsmit J, Danner SA, Haase AT (1997). Kinetics of response in lymphoid tissues to antiretroviral therapy of HIV-1 infection. Science.

[50] Warner N, Núñez G (2013). MyD88: a critical adaptor protein in innate immunity signal transduction. J Immunol.

[51] Du Y, Zhang L, Huang B, Guan X, Li L, Zhang G (2013). Molecular cloning, characterization, and expression of two myeloid differentiation Factor 88 (Myd88) in Pacific Oyster, Crassostrea gigas. J World Aquac Soc.

[52] Litwack ED, Ivins JK, Kumbasar A, Paine-Saunders S, Stipp CS, Lander AD (1998). Expression of the heparan sulfate proteoglycan glypican-1 in the developing rodent. Dev Dyn.

[53] Gonzalez AD, Kaya M, Shi W, Song H, Testa JR, Penn LZ, Filmus J (1998). OCI-5/GPC3, a glypican encoded by a gene that is mutated in the Simpson-Golabi-Behmel overgrowth syndrome, induces apoptosis in a cell line-specific manner. J Cell Biol.

[54] Cano-Gauci DF, Song HH, Yang H, McKerlie C, Choo B, Shi W, Pullano R, Piscione TD, Grisaru S, Soon S, Sedlackova L, Tanswell AK, Mak TW, Yeger H, Lockwood GA, Rosenblum ND, Filmus J (1999). Glypican-3-deficient mice exhibit developmental overgrowth and some of the abnormalities typical of Simpson-Golabi-Behmel syndrome. J Cell Biol.

[55] Khanobdee K, Soowannayan C, Flegel T, Ubol S, Withyachumnarnkul B (2002). Evidence for apoptosis correlated with mortality in the giant black tiger shrimp Penaeus monodon infected with yellow head virus. Dis Aquat Organ.

[56] Li MF, Traxler GS (1972). Antiviral activity of aqueous clam (Mya arenaria) extract on amphibian virus (LT-1). Can J Microbiol.

[57] Lee T-G, Maruyama S (1998). Isolation of HIV-1 protease-inhibiting peptides from thermolysin hydrolysate of oyster proteins. Biochem Biophys Res Commun.

[58] Renault T, Le Deuff RM, Cochennec N, Maffart P (1994). Herpesviruses associated with mortalities among Pacific oyster, Crassostrea gigas, in France-Comparative study. Rev Med Vet.

[59] Carriel-Gomes MC, Kratz JM, Müller VDM, Barardi CRM, Simões CMO (2006). Evaluation of antiviral activity in hemolymph from oysters Crassostrea rhizophorae and Crassostrea gigas. Aquat Living Resour.

[60] Zeng M, Cui W, Zhao Y, Liu Z, Dong S, Guo Y (2008). Antiviral active peptide from oyster. Chin J Oceanol Limnol.

[61] Dang VT, Speck P, Benkendorff K (2012). Influence of elevated temperatures on the immune response of abalone, Haliotis rubra. Fish Shellfish Immunol.

